# Supplementary material for: Splice-Junction-Based Mapping of Alternative Isoforms in the Human Proteome
Source: Cell Rep. Author manuscript; Available in PMC 2020 Jan 15. (PMC6961840; doi:10.1016/j.celrep.2019.11.026)
Supplement: 2 [file NIHMS1546469-supplement-2.zip › DF1/PXD010154/Ovary/Ovary_15_ADAMTSL3_HAAIMTALQMQK.pdf]

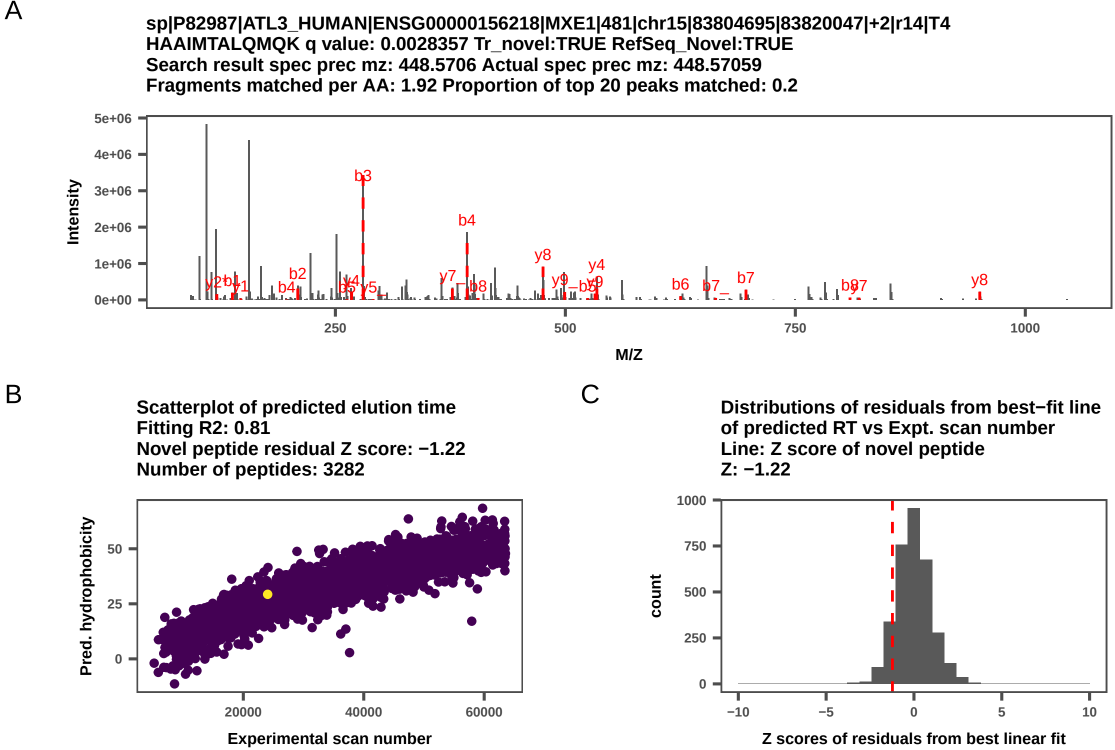
A
sp|P82987|ATL3_HUMAN|ENSG00000156218|MXE1|481|chr15|83804695|83820047|+2|r14|T4
HAAIMTALQMQK q value: 0.0028357 Tr_novel:TRUE RefSeq_Novel:TRUE
Search result spec prec mz: 448.5706 Actual spec prec mz: 448.57059
Fragments matched per AA: 1.92 Proportion of top 20 peaks matched: 0.2
Intensity
5e+06
4e+06
3e+06
2e+06
1e+06
0e+00
b3
b4
y8
y4
b2
b6
b7
y8
250
500
750
1000
M/Z
B
Scatterplot of predicted elution time
Fitting R2: 0.81
Novel peptide residual Z score: −1.22
Number of peptides: 3282
Pred. hydrophobicity
50
25
0
20000
40000
60000
Experimental scan number
C
Distributions of residuals from best−fit line
of predicted RT vs Expt. scan number
Line: Z score of novel peptide
Z: −1.22
count
1000
750
500
250
0
−10
−5
0
5
10
Z scores of residuals from best linear fit
